# Topics of the Month

**Published:** 1895-06

**Authors:** 


### TOPICS OF THE MONTH.

The New York Academy of Medicine held memorial exercises on Thursday evening, May 3, 1895, in recognition of the character and work of the late Dr. Alfred Lebbeus Loomis, a former president of the academy and one largely instrumental in building the present handsome structure in West Forty-third street.

Dr. Joseph D. Bryant, president of the academy, presided at the meeting, and addresses were made by Dr. Lewis A. Stimson, Chancellor Henry M. MacCracken, ex-Mayor Abram S. Hewitt, Dr. Reginald H. Sayre and others. Among the leading physicians of the city who attended the services were Drs. S. S. Purple, A. Jacobi, R. C. M. Page, Charles MacBurney, J. P. Munn, Virgil P. Gibney, W. H. Thomson, J. E. Winters, C. C. Rice, L. L. Seaman, M. H. Katzenbach, J. F. Erdman, A. E. Macdonald, W. M. Polk and W. Lester Carr. The family of Dr. Loomis were present by invitation, and many women were in the large audience.

Dr. Stimson, in his address, spoke of the fact that Dr. Loomis feared that he would not live until he was thirty, knowing that he had inherited consumption. Dr. Loomis, he said, was elected president of every society of which he was a member, and in every case the honor came to him unsought. He became a fellow of the Academy of Medicine in 1863, was elected a trustee in 1885 and was chosen president of the academy in 1889. He founded two hospitals for the treatment of consumptives in the country.

Chancellor MacCracken spoke of Dr. Loomis's work in the medical college and of the founding of the Loomis Laboratory in connection with the college. He also spoke of Dr. Loomis as a man.

Mr. Hewitt in his address spoke of the work which Dr. Loomis had performed as a citizen, and said that when he was mayor of the city he had consulted Dr. Loomis repeatedly about health matters.

---

The injustice done the medical corps of the U. S. Navy in according to the members inferior rank and pay, has long been known as a standing disgrace to the government. It is only second to that other scandal whereby junior officers are promoted over seniors—a method that deserves the execration of every intelligent American citizen. With a view to correct the former evil, Dr. P. H. Millard, of Minneapolis, introduced the following preamble and resolutions at the late meeting of the American Academy of Medicine in Baltimore, which received the unanimous approval of the academy:

*Whereas*, Membership in the medical corps of the Navy of the United States from having been, through its high professional attainments, once eagerly sought by graduates of medical colleges, has ceased to be attractive to them, so that it is no longer possible to fill its vacancies; and

*Whereas*, It is believed that this is due to the inferior rank given to entrants to the corps and their assignment, when at sea, to quarters in the steerage with undergraduates of the Naval Academy and civilian paymaster's clerks, while the young medical officer of the army begins his career a grade higher and in the commissioned officers' mess; and

*Whereas*, The professional requirements and qualifications, the professional duties and obligations of the medical officers of the army and navy are identical, therefore, be it

*Resolved*, That the attention of the Senate and House of Representatives of the United States be invited by the American Academy of Medicine to this inequality, and that the naval committees of the two houses be urged to inaugurate such legislation as shall confer upon the officers of the medical corps of the navy the same status, pay and emoluments as are now accorded to the medical officers of the army.

*Resolved*, That this resolution be printed and copies of it, certified by the president and secretary of the association, shall be sent to the President of the United States, the secretary of the navy and to every member of the Fifty-fourth Congress.

Similar action was taken by the American Medical Association a day or two later. We hope these two influential bodies will follow up their action by urgent demands until congress comes to the relief of the naval medical corps.

---

DR. ALBERT L. GIHON, medical director U. S. Navy, presented a report as chairman of the committee on the Rush monument at the recent meeting of the American Medical Association, which possesses many points of interest. The report regretfully stated that there was in the hands of the committee, at this date, only $3,094.39. Dr. Gihon said this was a reproach to the medical profession in this country and compared very unfavorably with the success of the American Surgical Association, which has collected all the funds necessary for the projected statue to the distinguished surgeon, Samuel Gross, of Philadelphia.

Dr. Gihon gave a brief sketch of Dr. Benjamin Rush, the soldier physician of the Revolution and signer of the Declaration of Independence, and paid an eloquent tribute to his patriotic services to the nation and the benefits he had conferred upon the profession. Dr. Rush died a victim of typhus-pneumonia, at the age of 68, while battling with the great epidemic of 1813. His portrait, by Sully, hangs in the reception-room of the Pennsylvania Hospital.

Dr. Gihon closed by saying that his committee had selected the site for the monument in the City of Washington, and he would guarantee that Congress would furnish the granite base or pedestal. The bronze statue with which it is proposed to surmount this is estimated to cost between $20,000 and $25,000.

Dr. H. D. Holton, of Vermont, arose during the applause that followed the reading of the report, and, warmly seconding the sentiments expressed by the latter, offered himself as one of a hundred members of the association to subscribe $100 each. His offer was closely followed by similar offers of $100 each on the part of Dr. W. H. Daly, of Pittsburg; Dr. John A. Wyeth, of New York; Dr. O. H. Wingate, of Wisconsin; Dr. H. O. Marcy, of Boston; Dr. J. M. Keller, of Arkansas; Dr. S. M. Free, of Pennsylvania; Dr. Jerome Cochran, of Alabama; Dr. Alonzo Garcelon, of Maine; Dr. J. M. Ridge, of New Jersey; Dr. I. N. Love, of St. Louis; Dr. B. D. Evans, of New Jersey; President Donald Maclean, of Michigan, and Dr. O. D. Will, of Peoria, Ill. Dr. J. M. Reeves, of Chattanooga, Tenn., and Dr. A. C. Cotton, of Chicago, gave $50 each, and Dr. Patrick Espy, of Norwich, Ct., gave $25. Dr. John A. Wyeth, of New York, offered to be one of six members to subscribe $500 apiece. The total subscriptions received during the day amounted to $2,257, including the smaller amounts subscribed after the meeting. A motion was passed that each member of the association act as a committee of one to solicit subscriptions for this fund from the whole medical profession of the country.

On motion of Dr. Daly, of Pittsburg, the thanks of the association were extended to Dr. Gihon for his work in behalf of the monument, the report of the committee was ordered to be published in full in the *Journal*, and the committee was continued.

---

THE American Medical College Association, at its recent meeting in Baltimore, took most important action looking to the establishment, in the near future, of four years' courses in all the colleges holding membership in the association. The differences existing between the Southern College Association and the National body were adjusted and an amalgamation of the two organizations effected.

The resolution advancing the standard to four years' study as a minimum, including collegiate training, was then unanimously adopted as above indicated. It ought not now to be difficult to obtain an act of the New York legislature rendering it unlawful for the medical colleges in this state to grant diplomas after less than four years' collegiate training.

---

THE *Montreal Medical Journal*, in a recent issue, makes the following appropriate comments upon an unjust tax :

> We believe that the Canadian Government stands alone among the governments of the civilized world in exacting a duty on the diphtheritic antitoxin. It is difficult to understand the short-sighted policy of instituting such a tax. We are informed that a few of the first importations of the diphtheritic antitoxin were admitted free, but that the officious officials decided that a duty of 50 per cent. should be levied in future.
>
> It is to be hoped that this and similar unjust taxes will soon be done away with. Now that the cabinet have a medical man among their number, this hope may be realized. The taxing, and that heavily, of nearly all instruments, appliances and apparatuses used in the investigation and treatment of disease is a matter that has been repeatedly brought to the notice of the Canadian Government, but without any result. Such a tax is a great hindrance to the advance of the profession. It is not only felt by practitioners in their private work, but also by hospitals. All suffer—the rich as well as the poor.

---

THE JOURNAL has, on several occasions, commented on the fog-horn nuisance to which Buffalo has been subjected during the past two years. There is no doubt that it has driven people out of the city who, otherwise, gladly would have remained here. Again, it has impaired the health of others by preventing sleep when most needed, and has in many other ways jarred and irritated over-strained nerves.

At last it seems we are in a fair way to obtain partial relief, at least, thanks to the kind offices of Hon. E. B. Jewett, our able and efficient mayor. A communication from him on the subject to the government at Washington brought quick response, and the lighthouse officials of this district are reported to have agreed upon a plan whereby a sounding-board is to be erected, and the whistle is to be blown less frequently—both of which will tend to modify, if not abate, the nuisance. Let us as a people exclaim to the light-house board and to Mayor Jewett especially: For this prospective relief, much thanks!

---

WE HAVE received the following clever verses that we print in this department of the JOURNAL, for, in the absence of our biologic editor, who attends to such details, we are at a loss just where to place them. However, like the celtic rabbit, they are good no matter when, where or how served:

(*Respectfully dedicated to our City Bug-hunter. By S.*)

Where schizomycetes grin in glee,
  Where microbes dig their holes,
Where germs feel free, to split in thee,
  And delight in their septic souls;
Where the small, still sobs of the spores endure
  And blue bacilli bore,
Where of cultures pure, an adjacent sewer,
  Will always furnish a score,—
Some certain cocci, duplex and small,
  (They were born at Woodlawn the previous Fall
And by agile turn and twisting flight
  Had escaped the maw of the phagocyte),
Were mourning each to the other twin—
"What sort of a place is this we're in?"

Time was when they lived where their fathers died;
  In a giant columnar cell,
Then woe betide that Devil they spied
  As they frisked in Bocaccio's hell.
Now, 'twas "Oh! for a lodge in the vestibule
  Or some other place near kin,
As was the rule in the Skene's tubule,
  For we can't grow in gelatine."
And 'twas "Oh! for the grim and fierce delight
  Of the duel to death with a leucocyte;
Once again to laugh in specific pride,
  At the impotent chemical and germicide,
And to mock at the organisms they defied
The bacilli, spirilli, side by side."

And 'twas "Oh! to infect the fresh, fresh youth
  And his giblets old and gray;
And the jay uncouth, as with Sal and Ruth
  He sports in the new mown hay."

And 'twas "Oh! to breed in the infant's eye
And make them greatly swell,
As the poor kid cried, the thing defied
It raised a merry—(WELL)!"
'Twas the same where'er they did go
They infected everything, nearly so;
Nary a spore grew weak or lean,
The venom departed as a new ptomaine,
And whatever spot they smote
No one found an antidote.

Let the staphylococcus laugh long and loud
At the sounds of the helpless wail,
Though our heads be bowed, no putrid crowd
Such honor as ours assail.
We've snapped many a streptococcus's chain,
Hushed many a bacillus's bray,
And his search be vain, who will look for a stain
On the record we hold today.
Come death to us, not a bit,
Excepting that storming, steaming pit,
And never will our words belie,
As long as our bug-hunter is handy by,
But neither in culture, in mucus, or pus,
Has Bissell, himself, found "flies on us."

